# Symmetry prediction and knowledge discovery from X-ray diffraction patterns using an interpretable machine learning approach

**DOI:** 10.1038/s41598-020-77474-4

**Published:** 2020-12-11

**Authors:** Yuta Suzuki, Hideitsu Hino, Takafumi Hawai, Kotaro Saito, Masato Kotsugi, Kanta Ono

**Affiliations:** 1 Institute of Materials Structure Science, High Energy Accelerator Research Organization (KEK), Tsukuba, Ibaraki 305-0801 Japan; 2 School of High Energy Accelerator Science, The Graduate University for Advanced Studies (SOKENDAI), Tsukuba, Ibaraki 305-0801 Japan; 3 The Institute of Statistical Mathematics, Tokyo, 190-0014 Japan; 4 Paul Scherrer Institute (PSI), 5232 Villigen, Switzerland; 5 Medley, Inc., Tokyo, 106-6222 Japan; 6 Department of Materials Science and Technology, Tokyo University of Science, Tokyo, 125-8585 Japan

**Keywords:** Structure of solids and liquids, Characterization and analytical techniques, Characterization and analytical techniques

## Abstract

Determination of crystal system and space group in the initial stages of crystal structure analysis forms a bottleneck in material science workflow that often requires manual tuning. Herein we propose a machine-learning (ML)-based approach for crystal system and space group classification based on powder X-ray diffraction (XRD) patterns as a proof of concept using simulated patterns. Our tree-ensemble-based ML model works with nearly or over 90% accuracy for crystal system classification, except for triclinic cases, and with 88% accuracy for space group classification with five candidates. We also succeeded in quantifying empirical knowledge vaguely shared among experts, showing the possibility for data-driven discovery of unrecognised characteristics embedded in experimental data by using an interpretable ML approach.

## Introduction

Crystal structure characterisation is one of the most important tasks in materials development because crystal structure determines material properties^1,2^. A crystal structure is defined in terms of lattice symmetry, lattice parameters, the types and positions of atoms, and site occupancy. Powder X-ray diffraction (XRD) and powder neutron diffraction are principal experimental techniques to elucidate crystal structures; data obtained using these techniques are stored in various databases for specific classes of materials, for instance, inorganic materials and proteins^3^. Decoding powder diffraction patterns to crystal structure information involves several steps, such as peak indexing, space group determination, initial parameter estimation for the crystal structure, and structure refinement^4–9^. While the most arduous step is structure refinement using the Rietveld method^10^, which typically requires manual optimisation of tens of parameters, space group determination at the initial stage of structure analysis also needs manual trial-and-error operations frequently. Given that a large number of powder XRD patterns are generated daily at synchrotron facilities around the world, these time-consuming processes performed manually by human experts are obvious bottlenecks in materials research^11–13^. Excluding human involvement in these processes as far as possible improves the situation and helps realise high-throughput (HiTp) experiments. Therefore, we focus on the classification of crystal systems and space groups using machine learning (ML) approaches, inspired by the fact that experienced researchers can guess the crystal system from a given diffraction pattern.

Application of ML and related techniques for diffraction data analysis is a hot research topic in recent times^13,14^. Among various subtopics such as pattern decomposition and phase identification^15–18^ cluster analysis and phase mapping^19–23^, similarity metrics for comparison of diffraction data^24–26^ classification of a crystal symmetry^27–33^, a paper by Park et al.^34^ is relevant to this work. Park et al. classified crystal systems and space groups by applying a convolutional neural network (CNN) to simulated powder XRD patterns. They achieved high classification performance despite data deterioration due to Poisson noise and instrumental resolution. However, the complexity of CNN, or deep neural network, makes it difficult to interpret its internal processes to extract meaningful insights. While most materials informatics (MI) studies aim high classification or prediction accuracy, we believe that maintaining the possibility of data-driven knowledge discovery by using a human-interpretable model is important as well. If an ML model can classify the crystal class and the space group based on a diffraction pattern, it must have classification rules. By analysing the model, we could quantitatively specify the rules of thumb that experienced researchers have.

**Figure 1:**
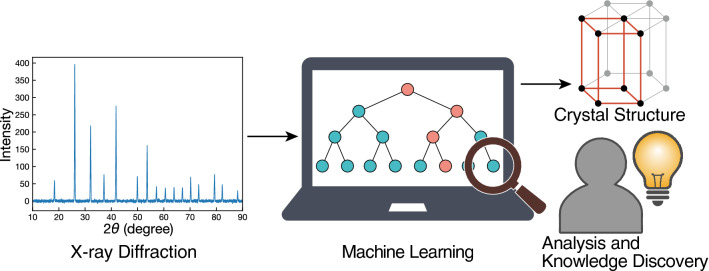
Overview of our ML methodology for crystal system and space group classification based on powder XRD patterns. One may also obtain data-driven insight by analysing the interpretable ML model.

In this paper, we show that a simple and fast ML technique (Fig. 1) can classify crystal systems (seven classes) and space groups (230 classes) with high accuracy based on powder XRD patterns and that data-driven quantification of empirical expert knowledge is possible using an interpretable ML model. We emphasise that the purpose of this work is neither to replace conventional methods nor to achieve state-of-the-art accuracy among other emerging ML-based techniques but to demonstrate the potential of simple ML techniques suitable for knowledge discovery and real-world experiments. Although this study is in a proof of concept (POC) stage using ML models trained on ideal simulated diffraction data (i.e. noise-free, no peak-broadening, no peak superposition, no impurity peaks), it provided some interesting findings described in following sections.

## Results

### Data preparation and feature extraction

**Figure 2:**
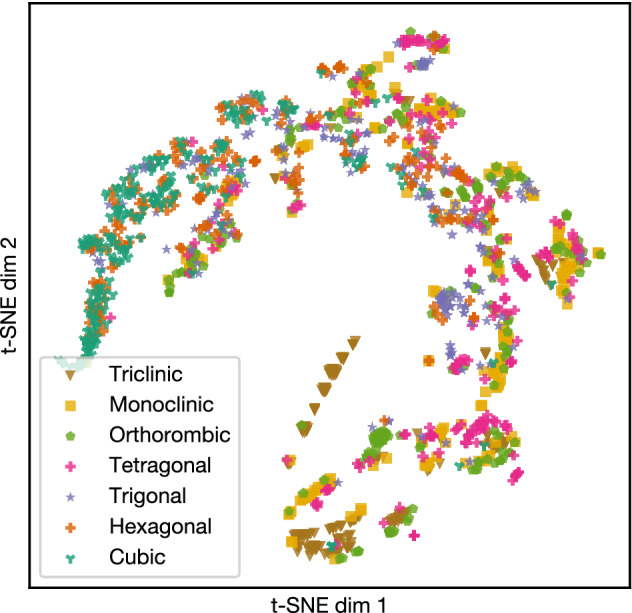
t-SNE visualisation of XRD patterns. Each point corresponds to one XRD pattern. XRD patterns form loose clusters for each crystal system.

199,391 powder XRD patterns were calculated as training datasets (see “Methods” section for details) from Inorganic Crystal Structure Database (ICSD) entries using Pymatgen middleware. The patterns were not used as they were for the following reason. Typical powder XRD patterns have several thousand data points, and, if used as they are, ML models treat them as several-thousand-dimensional vectors. Training an ML model using such extremely high-dimensional data inevitably suffers from “the curse of dimensionality”^35^, the fact that the amount of data required for training increases exponentially with increasing data dimensions. To avoid this problem, we reduced the thousands of data points into a handful of numbers characterising each XRD pattern. These quantities are usually called “features” or “descriptors”^13^, and we use the former term in this paper. In areas where a large amount of quality-controlled data is available, such as ImageNet dataset with about 14 million labelled images used for image recognition^36^, it has become mainstream to build feature extractors simultaneously with classification models by training deep neural networks with the huge dataset^36–38^. However, such approach is often inappropriate in MI due to two reasons: a limited amount of available data and difficulty in data quality control. In our case, while low quality data are eliminated as much as possible the data size is roughly two orders of magnitude smaller than ImageNet. Additionally, automatically-selected features are not always in a simple form for humans to understand. Therefore, we chose to select features manually using human expert knowledge. An XRD pattern (Fig. 1 left) has many peaks that together act as a fingerprint of a crystal structure. Among the peak characteristics, intensity (height) is mostly determined by the atomic positions and with a few exceptions, not to symmetry of crystal, which is the target of this study. Therefore, the following eleven features were selected for this study: (1) the positions of the first ten peaks in the lower-angle range. (2) the total number of peaks in $2\theta$ range from $0^\circ$ to $90^\circ$. Considering only the lower-angle peaks and ignoring the rest is justified because the peaks in the higher-angle range heavily overlap in real experimental data and it is impossible to identify individual peak positions, especially for low-symmetry cases. To examine the feature space, we have used Stochastic Neighbour Embedding with t-distribution (t-SNE)^39^, which is a popular method to visualise multi-dimensional data on a two-dimensional plane, preserving the local structure in the original multi-dimensional space. Figure 2 shows the result of the dimensionality reduction of eleven-dimensional data to two-dimensional space using t-SNE. The XRD patterns are distributed in the feature space and form loose clusters for each crystal system, implying that eleven features capture the characteristics of the XRD patterns at least in terms of crystal systems.

Here we mention ambiguity in the unit for peak positions. In most diffraction experiments, the horizontal axis of a diffraction pattern describes $2\theta$, which is the angle between the incident beam and the scattered beam. Through Bragg’s law $2d \sin \theta = \lambda$ and the definition of scattering vector length $Q = 4\pi \sin {\theta }/\lambda$ where *d* and $\lambda$ represent the separation of scattering planes and the wavelength of incident beam, respectively, $2\theta$ is occasionally converted to *d*, 1/*d*, $1/d^2$, or *Q*. We investigated the influence of these peak position notations on the classification accuracy and found that the difference is negligible (Table 1). Thus, herein we use $2\theta$ for Cu $\hbox {K}_{\alpha }$, which is the most commonly measured variable, and hence, the most familiar for many readers. It should be noted that the proposed method is applicable to any XRD patterns with arbitrary wavelength using wavelength-independent expressions above.

**Table 1:** Comparison of crystal system classification accuracy for various peak position notations and ML algorithms (in %).

Algorithm	Acc. ($2\theta$)	Acc. (*d*)	Acc. (1/*d*)	Acc. ($1/d^2$)	Acc. (*Q*)
Random forest	91.53	91.40	91.39	91.47	91.47
Extremely randomised trees	**92.24**	**92.16**	**92.22**	**92.17**	**92.26**
K-nearest neighbor	92.01	91.96	92.04	91.58	92.04
Decision tree	86.44	86.41	86.39	86.54	86.40
Logistic regression	56.00	57.80	56.14	55.22	55.97
CNN (10$^\circ$–110$^\circ$)^34^	94.99	–	–	–	–

Accuracy is a ratio of correct classification to all classification for the test data. Notably, the accuracy of CNN taken from Ref.^34^ is only for reference purpose because the dataset preparation and the evaluation condition were different from those used herein.

### Classification of crystal systems and space groups

Even though it is a POC, it is favorable to choose a algorithm satisfying some realistic requirements such as fast training speed, easy hyperparameter tuning, and an ability to provide multiple candidates so that researchers can tailor the model to meet their experimental conditions such as $2\theta$ range and wavelength, and choose the most reasonable candidate with the help of supplemental information.

Among various ML algorithms for classification tasks, random forest (RF) and its related algorithms satisfy all requirements mentioned above^40^. Table 1 shows the performance comparison of RF and other representative ML algorithms (Extremely randomised trees (ExRT)^41^, k-nearest neighbour (KNN)^42^, logistic regression^43^, and decision tree^44,45^) on the crystal system classification task. ExRT is an RF-based ML algorithm in which variables used for decision-making are chosen randomly in contrast to the significance-based selection criterion in normal RF. This randomness in ExRT reduces overfitting to the training data and improves the classification performance for unknown data. Additionally, we note the robustness of majority-voting algorithms including ExRT and RF. In actual experiments, some weak peaks are hard to recognise, or we may miss peaks outside the measurement range. Such deficiencies may cause wrong decisions for some of individual decision trees, but a majority decision taken by an ensemble of trees is often insensitive to such perturbations. Following the comparison result, we choose ExRT because it satisfies the requirements and exhibits the best performance. The computation time for the model training was a few minutes, and the classification for one XRD pattern only took several milliseconds with a general workstation (3.3 GHz 10-core CPU and 96 GB RAM), showing that the entire process can be performed even on an ordinary laptop computer within a reasonable time length.

**Figure 3:**
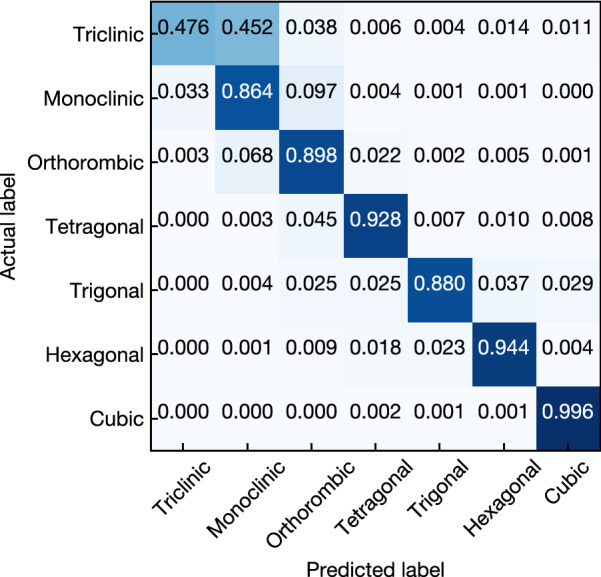
Confusion matrix for crystal system classification.

Classification performance for each crystal system with test dataset is shown in Table 2 and Fig. 3. Values in Fig. 3 represent the ratio of a crystal system classified by the ExRT model (predicted label) to a given crystal system dataset (actual label). The values along the diagonal correspond to the accuracy. Our model succeeded in classifying crystal systems with accuracy about 90%, which is higher than our expectation, except for triclinic cases.

**Table 2:** Classification performance of crystal system classification with ExRT in the test set.

Crystal system	Accuracy (%)	Precision (%)	Recall (%)	F1-score	Number of data
Triclinic	47.61	74.55	47.61	0.5811	1,403
Monoclinic	86.38	80.88	86.38	0.8354	5,895
Orthorhombic	89.84	86.91	89.84	0.8835	7,719
Tetragonal	92.77	92.73	92.77	0.9275	5,949
Trigonal	88.00	93.36	88.00	0.9060	3,532
Hexagonal	94.44	95.77	94.44	0.9510	6,093
Cubic	99.60	98.77	99.56	0.9918	15,477
Average	92.24	92.16	92.24	0.9209	(Total) 46,068

The low accuracy for triclinic cases may be attributed to the rarity of the triclinic structure in ICSD (only 4%), which hinders the classification.

**Figure 4:**
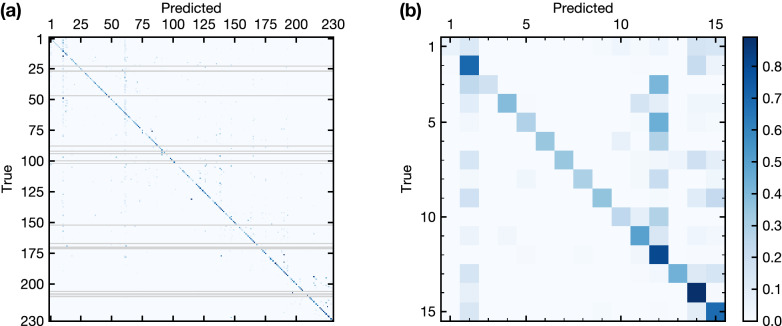
Normalised confusion matrix for space group classification. Normalisation is done in the horizontal direction. Tick labels represent space group numbers. (**a**) The overall confusion matrix. Some space groups lacked in the test data are shown as grey horizontal lines. (**b**) The confusion matrix of triclinic and monoclinic crystal systems [the upper left corner of (**a**)].

Along with the crystal system, we evaluated the performance of the space group classification as well. The classification accuracy of the most likely candidate proposed by the model was 80.46% (Fig. 4a). Considering that the number of training data for each space group is considerably smaller than that for each crystal system, this accuracy significantly lower than that of the crystal system classification is reasonable. However, if we consider a list of multiple candidates proposed by our model, the accuracy defined as the probability that the list includes the correct answer increases to 92.42% and 94.35% for five and ten most likely candidates, respectively. These values are more relevant to actual space group determination tasks compared to the accuracy with a single candidate because the multiple candidate approach is often applied in conventional space group determination methods as well.

In Fig. 4a, besides the diagonal line indicating the correct prediction, there are other noteworthy features and we conclude that two major causes showing the shortcomings of our model and one trivial cause are responsible for those features. First, we point out the trivial and less important one. There are some isolated non-diagonal thick blue pixels [e.g. (50, 12), (97, 139), (132, 116) in (row, column) notation]. These are false predictions of space groups with a few test structures deceptively emphasised by normalisation with the number of test data. Next, we describe the one of the two major cause, that is, overfitting, a common issue in machine learning. The overfitting issue is visible as thicker blue pixels aligned vertically at some space groups (2, 12, 14, 15, 62, and 139 in Fig. 4a and b), which means our ML model tends to pick up specific space groups for prediction. This issue is caused by the uneven distribution of space groups in the ICSD data used for training shown in Fig.  S1 in Supplementary Information as the histogram of our training data, that is, our model is biased to give an answer from what it have learnt frequently. The second major cause is an inherent defect in our approach itself which attempts to determine space groups only from the number and positions of peaks. To determine whether a crystal structure is centrosymmetric or not from a diffraction pattern, peak intensity is essential. Therefore, our ML model which ignores the peak intensity is intrinsically unable to determine the existence of centrosymmetry from a diffraction pattern.

Careful examination of the confusion matrix allows us to find some traces of this limitation. For example, in Fig. 4b, noncentrosymmetric space groups 1, 4, 6, 8, and 9 in test data are frequently predicted as their centrosymmetric minimal isomorphic supergroups 2, 11, 10, 12, and 15, respectively. Statistics also indicates the significance of this limitation in single-candidate prediction, that is, about 30% of false predictions (6% of test data) occur for centro- and noncentrosymmetric pairs.

We note that the identifying some false predictions as the centrosymmetric/noncentrosymmetric misclassification issue is exceptionally straightforward which is theoretically evident. For other misclassified results that are not relevant to centrosymmetry issue, we conclude that it is difficult to analyse the reasons from crystallographic points of view due to potential superposition of multiple causes mentioned above as well as the luck of our experience in such type of analysis.

Since there is a hierarchical relationship between space groups and crystal systems, the performance of space group prediction may be improved by using predicted crystal systems as the features for space group prediction. This idea to combine multiple ML models is called stacking^46^. Aguiar et al.^32,33^ reports that stacking improves the prediction accuracy for space group determination by electron diffraction. However, for our case, stacking did not improve the performance, i.e., the prediction performance was deteriorated. A possible reason is that misidentification in the crystal system prediction negatively affected the learning of the space group predictor as noise.

### Classification performance for new materials and actual experimental data

We perform two types of tests to assess the efficacy of our model on crystal structure classification tasks for new materials. The first one, whose results are already shown in Table 2, uses the powder XRD patterns calculated from a subset of ICSD. The subset consists of materials not included in the training datasets, that is, crystal structures unknown for the model (see “Methods” section for details). This test represents the performance of the model against new materials. As evident from Table 2, our model scores satisfactory performance except for the triclinic system, which is intrinsically difficult because of the lack of the triclinic structure data in ICSD as previously discussed.

So far, we have used only simulated patterns which are not deteriorated by statistical and experimental noises. The second test is meant to assess the model in a more practical situation. Instead of calculated peak positions, we use peak search results of two actual measured XRD patterns of $\hbox {Ca}_{1.5}\hbox {Ba}_{0.5}\hbox {Si}_{5}\hbox {N}_{6}\hbox {O}_{3}$ and $\hbox {BaAlSi}_{4}\hbox {O}_{3}\hbox {N}_{5}{:}\hbox { Eu}^{2+}$ taken from supplementary tables in Ref.^34^. We chose these two materials because their structures are so unique that they belong to none of over nineteen thousand prototype structures in ICSD as described in Ref.^34^. The auto peak search was performed in a conventional manner according to the explanation by the authors, which means using its results is practically equal to starting from raw XRD patterns that we do not have in our hands. Due to the limited 2$\theta$ range shown in the tables, we could not obtain the number of detected peaks suitable for our model. With this situation, we retrained our model without the total number of peaks and used only the ten lowest peak positions for classification. Nonetheless, the retrained model correctly classified not only the crystal systems of both compounds but also the space group of $\hbox {BaAlSi}_{4}\hbox {O}_{3}\hbox {N}_{5}:\hbox {Eu}^{2+}$. The CNN model in Ref.^34^ did succeeded in crystal system prediction for both compounds, but failed in space group prediction for both.

We analysed these benchmark materials with several standard peak-indexing programs in Crysfire2020 (ITO^47^, FJZN^48^, TREOR^49^, KOHL^50^, DICVOL^51^, LZON^48^) as well. However, none of them were able to give proper indexing results for both materials with the default settings. The comparison result is shown in Table S1 in the supplementary material. In Ref.^34^ the two materials were analysed with TREOR, and the authors of the paper also concluded that TREOR could not give proper results without human intervention. This fact indirectly shows that, at least for these specific cases, our method is more suitable for crystal system and space group estimation tasks than an existing software if less human intervention is preferred. Nonetheless, this comparison is not intended to claim superiority of our method. While both take peak positions as input, the type of output is different: the primal purpose of conventional peak-indexing programs is to give lattice constants and our method aims to estimate only crystal systems and space groups. This difference means that our model cannot be a substitute for long-lived existing peak-indexing programs.

Although the test size is extremely limited (only two) and our model failed one of the two space group prediction tasks, it is still worth noting that our model with only ten features gives a better result in space group prediction than the CNN model using ten thousand data points as features which failed for two test compounds. This indicates that appropriate feature selection is important for this task. Additionally, the success of the ExRT model trained without the total number of peaks may broaden the target range of our approach because some XRD patterns are not suitable for correctly counting the number of peaks because of impurity peaks, the bad signal-to-noise ratio, or severe peak overlapping.

### Knowledge extraction from machine learning models

In terms of prediction performance, there was no significant difference between KNN and ExRT as shown in Table 1. However, tree-based models such as ExRT and decision tree provide more information to us than KNN. That is, our ML model may provide an insight into the relevant tasks as an additional advantage through careful analysis. Such knowledge may help in designing a minimum but effective measurement setup and an experiment plan for a specific purpose.

In this regard, we investigate the following points to exploit our ML model: (1) evaluation of feature importance, (2) classification performance dependency on the number of features (peak positions), (3) quantification and visualisation of decision rules for crystal system classification.

Table 3 shows the feature importances of eleven features used in our model for the crystal system classification task. Feature importance quantifies the contribution of a feature to the improvement of the splitting quality. We use Gini impurity^46^ as a split criterion in this study.

**Table 3:** Feature importances for crystal system classification. Peak *n* represents the position of *n*-th lowest angle peak.

Feature	Number of peaks	Peak 1	Peak 2	Peak 3	Peak 4	Peak 5	Peak 6	Peak 7	Peak 8	Peak 9	Peak 10
Importance	0.184	0.109	0.089	0.095	0.094	0.084	0.070	0.069	0.063	0.070	0.073

Larger importance on the number of peaks and peak positions at lower angles can be interpreted as follows. Because most lattice constants of the crystal structures stored in ICSD are available within a certain range, the main factor that determines the number of peaks within a 2$\theta$ window that we use is the symmetry of a crystal structure. Similarly, under the same experimental condition, the positions of the ten lowest-angle peaks primarily depend on the extinction rule determined by the symmetry of the structure. For example, simple cubic structures whose symmetry is the highest among the seven crystal systems have three reflections, 001, 011, and 111 in this order in the lowest $2\theta$ range. If the structure changes from cubic to orthorhombic, both 001 and 011 reflections of the original cubic structure split into three reflections, and in some cases, even high-order reflections appear in the lower $2\theta$ side of the 111 reflection. As a result, most of the ten lowest-angle peaks are dominated by such split peaks and their positions shift to a lower $2\theta$ range, compared to the cubic case. A similar argument is also valid for general cases related to the comparison between two crystal systems not involving a structural phase transition. Therefore, if many peaks appear in the low $2\theta$ region, it implies that the symmetry is low and vice versa. These inferences are consistent with the first impression of experts. Notably, these inferences could be extracted quantitatively from the ML model, which we will discuss later.

**Figure 5:**
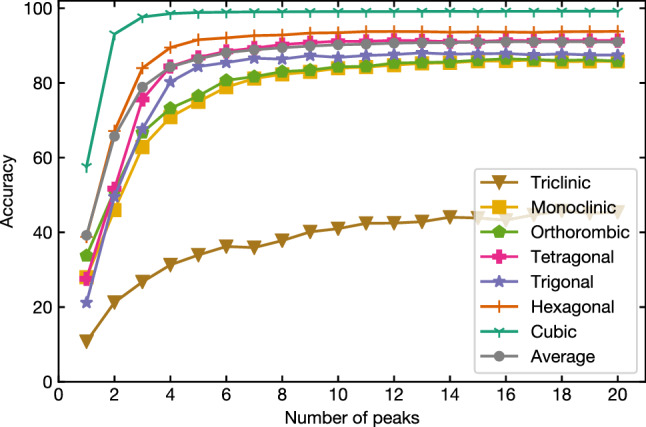
Classification accuracy versus number of peak positions used in training.

Next, we evaluate the importance of lower peaks in the actual classification task by plotting the dependency of classification accuracy on the number of peak positions used in model training (Fig. 5). Here, the total number of diffraction peaks was omitted from the features to extract the effect of peak positions. The accuracy saturates at five or six lowest peak positions for most of the crystal systems, and adding higher peak positions in training only gives a marginal improvement. Here, the qualitative tendency was revealed that more complex crystal systems require more information for identification.

As discussed in the Introduction, experienced researchers can estimate the crystal system from a given XRD pattern without any additional information, mostly when the symmetry of the crystal structure is high. In general, such expert knowledge obtained through experience is vague and difficult to quantify. Here, we present an attempt to quantify expert knowledge on a simplified crystal system classification task, i.e. binary classification of cubic and non-cubic systems.

**Figure 6:**
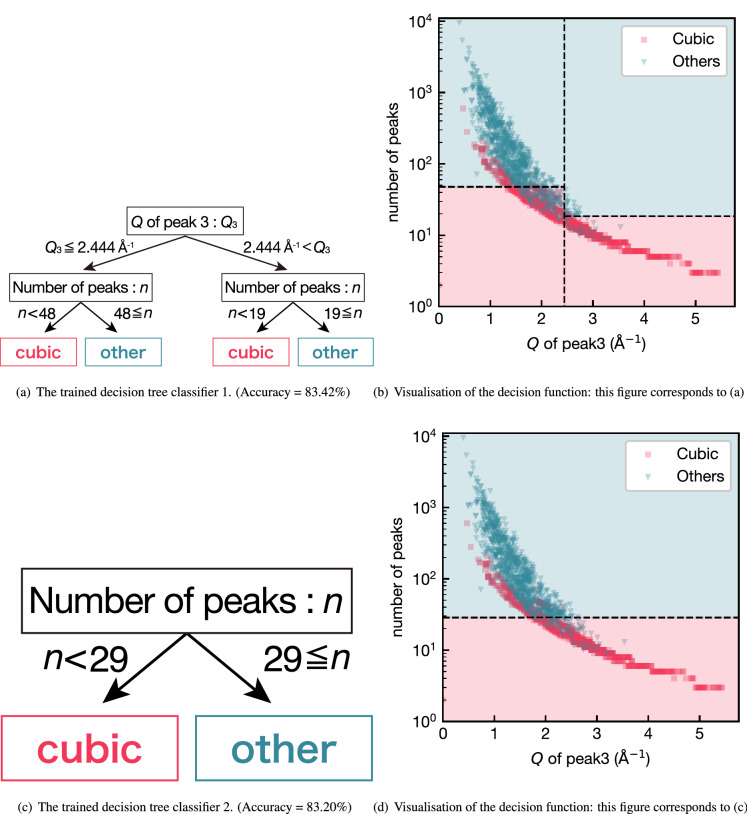
Visualisation of the decision tree classifier for cubic system identification. The ML model was trained with $2\theta$ features (see in “Data preparation and feature extraction” section). In this figure, the peak position is represented by the length of the scattering vector *Q* for wavelength-independency.

Because classification results by ExRT are based on the majority vote of several hundred decision trees with their maximum depth as a hyperparameter, it is difficult to extract the classification rules from our ExRT model in a reasonable way. Instead, we repeatedly train a single decision tree for a binary classification task (cubic or non-cubic system) and a crystal system classification task (seven classes) with different random seeds. Two representative decision trees and their splitting rules for the binary classification are shown in Fig. 6.

The splitting rules of the two trees consist of only two features in total, and the accuracy is surprisingly high despite such simple tree structures of depth one or two. The number of peaks, which is employed by both trees, is a reasonable criterion to distinguish cubic and non-cubic crystal structures because higher symmetry crystal structures have less Bragg peaks in their diffraction patterns in general. On the contrary, it is not as straightforward as the case for the number of peaks to interpret how the other criterion, the position of peak 3, contributes to the binary classification task. A hint is found in Fig. 6b and d where the splitting rules are visualised in a scatter plot, i.e. the wider distribution of data points for the cubic system along the horizontal axis representing the position of third lowest-angle peak (peak 3). As already discussed, the feature importance indicates that lower-angle peak positions are more important in the crystal system classification task. The difference in the distribution of peak 3 between cubic and non-cubic systems implies that an effective threshold exists for the binary classification task, which is the exact strategy that the decision tree in Fig. 6a takes. This argument can be regarded as a case showing the importance of lower peaks for a specific task, that is “cubic-or-not” classification, and also an example of quantitative knowledge extraction using an ML model.

We confirmed the similar trend in the crystal system classification. The decision tree is visualised in Fig. S3 in the supplemental material.

## Discussion

The highlight of this study is quantitative knowledge extraction enabled by the analysis of a trained ML model, which is hard to realise only with physical principles. We expect this idea is relevant to experimental setup optimisation because many experiments in material science still rely on conventional rules-of-thumb parameters. For example, experimental parameters such as scanning ranges, data point intervals, and measurement time can be objectively tailored for a specific purpose if an ML model indicates guidelines for these parameters to assure minimal but sufficient data quality for the purpose. An efficient experimental design is particularly crucial for experiments using synchrotron X-rays and neutron beams, where the efficient use of the measurement time is essential because of limited beamtime available^52–54^.

Another highlight is that our ML model does not require large computational resources: training two ExRT models for crystal system and space group classification tasks will only take a few minutes on a modern laptop computer in contrast to the CNN model proposed in Ref.^34^ which requires more than a day for training even with one of the currently fastest GPUs (Nvidia TITAN RTX). This advantage is crucial for on-site customisation because measurement parameters such as wavelength and $2 \theta$ range may vary in each diffraction experiment and sample.

A noticeable flaw in our model, that is, the low accuracy for non-cubic systems, especially for triclinic cases, presumably stems from the shortage of training data in these crystal systems. This type of problem is called the class imbalance and is resolved by a technique called “data augmentation” in some fields in ML such as image and speech recognition tasks^37,38,55,56^. Data augmentation for a crystal-system learning task is theoretically possible in a straightforward way as well; by distorting or expanding actual crystal structures, we can generate any number of artificial low-symmetry crystal structures. However, due to the enormous amount of the possible combinations of artificial structures, the improvement of the classification accuracy using data augmentation is beyond the scope of this paper and left as a future issue.

The scope of this paper is limited to proposing a new method using ML, and we do not expect our current models to work well with real data at this moment. However, it is interesting to check the current performance of our approach for real-world data, so we did some tests. Please refer to “Classification performance for new materials and actual experimental data” sectiion in the main text and Section S4 in Supplementary Information. To apply this method to real-world data, the ML model should be flexible about imperfections such as peak overlap and impurity peaks under practical measurement conditions, but we did not address these issues at this moment. In future work, we will update our models and verify them on real experimental data.

## Methods

### XRD datasets

XRD patterns were generated from the crystal structures registered in ICSD (2018.1) using Pymatgen middleware. The X-ray wavelength was set to 1.54184 Å (Cu $\hbox {K}_{\alpha 1}$), and the $2\theta$ range was $0^\circ$ to $90^\circ$. It is well known that removing “bad data” from training data is crucial to avoid detrimental effects on a trained ML model^57^. We excluded about 30k structures exhibiting one of the following issues:

1. Missing information (i.e., null in the atom position): 19967 samples.
2. Mismatch in space groups between those calculated from the crystal structure using spglib^58^ and those registered in ICSD: 8540 samples. One of the possible reasons for the mismatch is rounding because some of these structures have 0.67 in one of atomic positions, which is close to a special value 2/3. We chose not to investigate the cause of the mismatch in detail because it is difficult to investigate over 8000 samples whether the disagreement is due to wild rounding or a valid shift from a special value. We simply excluded all structures showing this space group mismatch. We note that the impact of this exclusion on the hexagonal structures is limited. The number of excluded hexagonal structures due to this space group mismatch is 2067, which corresponds to 9.0% of 22,953 hexagonal structures registered in ICSD.
3. R factor larger than 20%: 948 samples. Large R factor implies that the refined crystal structure is not reliable.
4. Huge lattice constants (> 50 Å): 1022 samples. These structures are exceptionally complex and seem to be approximated expressions for amorphous materials. They are inappropriate for our purpose because we do not focus on amorphous materials and such outliers deteriorate ML models.
5. Tiny lattice constants (< 2.5 Å): 173 samples. Assumed to be crystal structures under high pressure. These structures have only a few Bragg peaks and are inappropriate likewise.

In all, 169,563 XRD patterns were used in this study. The distributions of crystal systems in ICSD and reduced dataset is shown in Figs. S1 and S2 in the supplementary information.

For generalisation performance evaluation, we employed a date-based data-splitting policy instead of the conventional random data-splitting policy to reduce the possibility of data leakage. As a nature of materials science research, there are many systematic works reporting a series of compounds with different compositions showing almost identical crystal structures. If we choose crystal structures randomly, the test data likely contains a certain amount of structures from those works, which results in data leakage. We expect this issue is partly alleviated by splitting the database with a chronological threshold. In our case, the data registered in ICSD until 2014 (123,495 samples) were used as the training data, and those registered after 2014 (46,068 samples) were used as the test data. We confirmed that both datasets have a similar composition of crystal classes.

A non-cubic dataset for binary classification in Fig. 6 was undersampled so that the numbers of cubic structures and non-cubic structures were equal, and the prediction performance of the trained model was evaluated with 10CV as well. For the visualisation of crystal system classification rules (Fig. S2), the decision tree was trained using the randomly sampled dataset to include 2000 entries of each crystal system from the training data. The prediction performance of the decision tree was verified using the test data.

### Machine learning models

The following ML algorithms were employed in this study: Logistic Regression^43^, K-Nearest Neighbour^42^, Decision Tree^44,45^, Random Forest^40^, Extremely randomised trees^41^. These algorithms are available in scikit-learn^59^. Hyperparameters of ML models were determined by random search, and the classification performance was evaluated using 10-fold cross-validation (10CV) with the training data. The parameter showing the best performance was used in this study.

For Logistic Regression, feature standardisation, which makes the values of each feature in the data have zero-mean and unit-variance, was performed to standardise the range of independent features.

For the classification performance measure, we use accuracy, recall, precision, and F1-score^60^. The first three measures are defined as follows with the number of true positive (*TP*), true negative (*TN*) false positive (*FP*), and false negative (*FN*) predictions:

1 $$\begin{aligned} \mathrm {Accuracy}&= \frac{TP+TN}{TP+FP+TN+FN} \end{aligned}$$

2 $$\begin{aligned} \mathrm {Precision}&= \frac{TP}{TP + FP} \end{aligned}$$

3 $$\begin{aligned} \mathrm {Recall}&= \frac{TP}{TP + FN} . \end{aligned}$$

And using precision and recall, F1-score is defined as follows:

4 $$\begin{aligned} \mathrm {F1} = 2 \times \frac{\mathrm {Precision} \times \mathrm {Recall}}{\mathrm {Precision} + \mathrm {Recall}} \end{aligned}$$

## Supplementary information

Supplementary Information.

## Data Availability

The dataset and codes that support the findings of this study are available at https://github.com/quantumbeam/xrd-symmetry-prediction.
