# Supplementary material for: Symmetry prediction and knowledge discovery from X-ray diffraction patterns using an interpretable machine learning approach
Source: Sci Rep. 2020 Dec 11;10:21790. doi: 10.1038/s41598-020-77474-4 (PMC7732852; doi:10.1038/s41598-020-77474-4)
Supplement: Supplementary file 1 — Supplementary Information. [file 41598_2020_77474_MOESM1_ESM.pdf]

Supplemental material for Symmetry prediction and knowledge discovery from X-ray diffraction patterns using an interpretable machine learning approach

Yuta Suzuki[1,2], Hideitsu Hino[3], Takafumi Hawai[1], Kotaro Saito[4], Masato Kotsugi[5], and Kanta Ono[1,2*]

[1]High Energy Accelerator Research Organization, Institute of Materials Structure Science, Ibaraki, 305-0801, Japan
[2]The Graduate University for Advanced Studies (SOKENDAI), School of High Energy Accelerator Science, Ibaraki, 305-0801, Japan
[3]The Institute of Statistical Mathematics, Tokyo, 190-0014, Japan
[4]Paul Scherrer Institute, 5232 Villigen PSI, Switzerland
[5]Tokyo University of Science, Department of Materials Science and Technology, Tokyo, 125-8585, Japan
[*]kanta.ono@kek.jp

1 The distribution of data classes in training data

Here, we show class population of our training data in Fig. S1 and S2. As mentioned in the main text, these histograms show an imbalance of the number of data in each classes in crystal systems and space groups. These two figures show that data reduction have no significant effect on the overall balance of distribution.

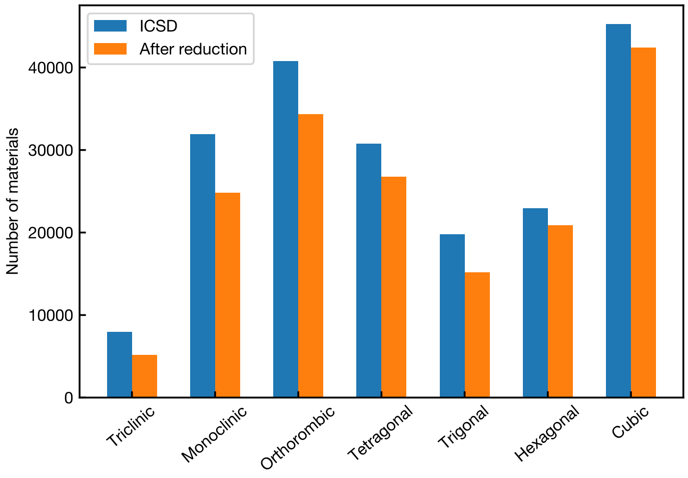

Figure S1: Histograms of crystal systems in our dataset before and after data reduction.

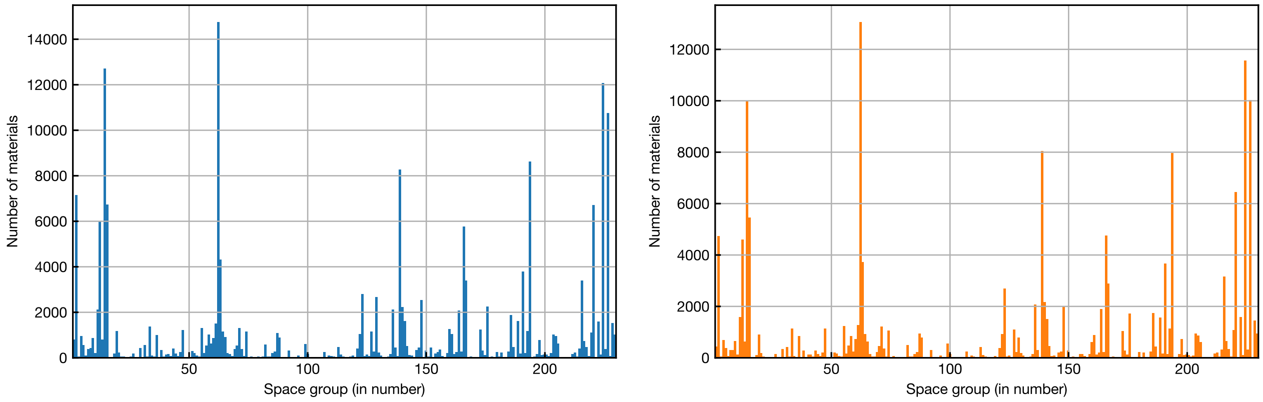

(a) ICSD. (b) After reduction.

Figure S2: Histograms of space groups in raw ICSD (a) and after reduction which used in this study (b).

2 Visualisation of the decision tree classifier for crystal system

We trained a simple decision tree for crystal system prediction, and is shown in Fig. S3. In this tree, there is a similar trend to Fig. 6 in the paper, which if there are few peaks and the ten lowest-angle peaks are at relatively high angles, they correspond to highly symmetric crystal structures and vice versa. Despite its ultimate simpleness, using one tree, we got an accuracy of 48.87 % for the test data. A specific examination of the predictions made by this model shows that the correct answer is only possible for a specific group of similar materials (e.g. Heusler alloys). This result illustrates the importance of the variety in decision trees for more accurate classification, as we did with ExRT.

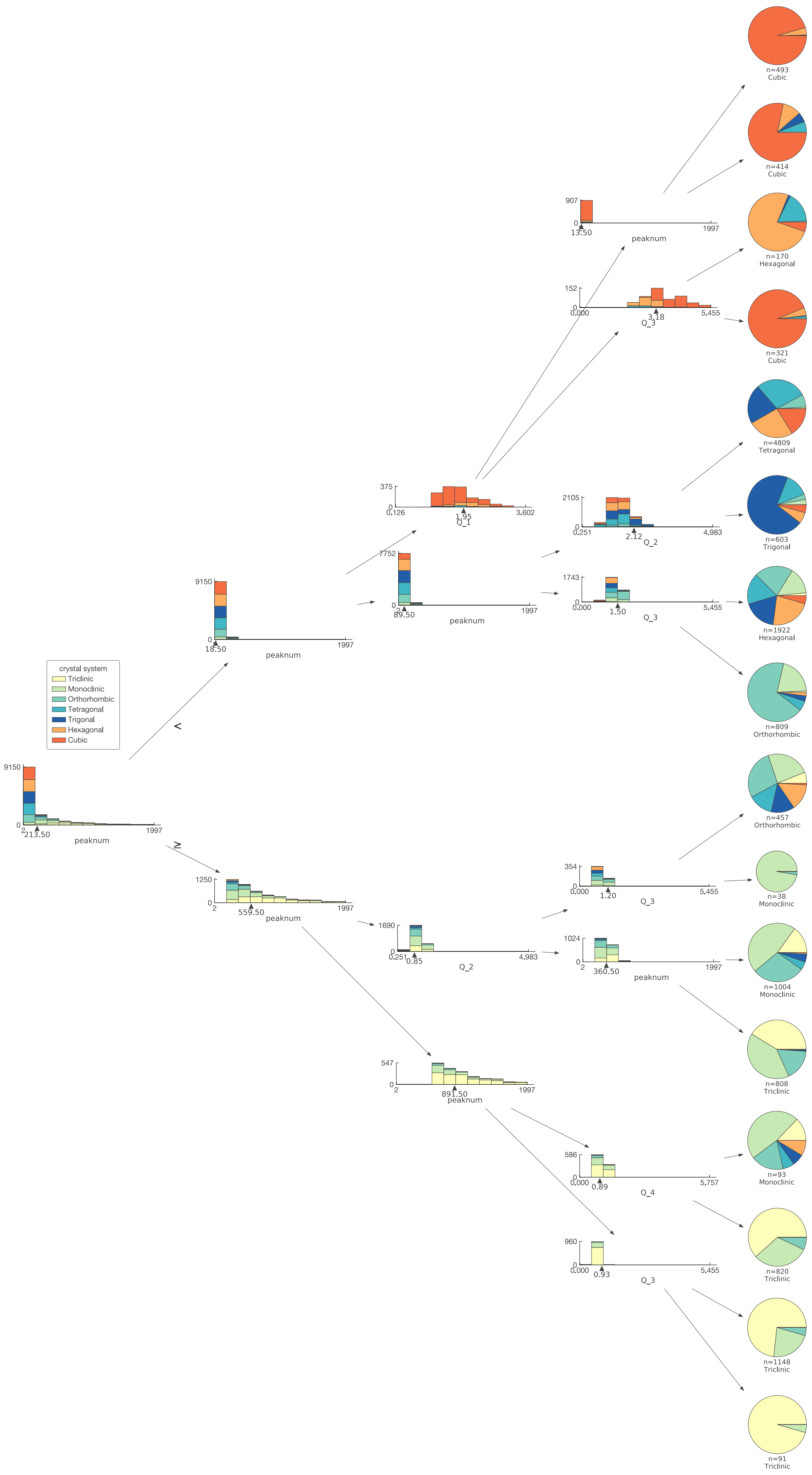

Figure S3: The trained decision tree classifier for crystal system prediction (Accuracy = 48.87 %). Pie charts and histograms represent the ratio of the crystal system in each node. The class name at the bottom of each pie chart indicates the majority class at the node, i.e., the prediction at the node. The thresholds of each splitting of the node are indicated in the histograms.

3 Comparison with conventional indexing software

3.1 Comparison on Park's benchmark materials

In Section 2.3 of the main manuscript, two materials ($Ca_{1.5}Ba_{0.5}Si_5N_6O_3$ and $BaAlSi_4O_3N_5$:Eu^{2+}) provided as Supplementary Information (Tb.S2 and S3) in the paper by Park *et al.* were used as benchmark materials for crystal system and space group classification. The indexing results of the two materials using standard peak-indexing software are shown in Tb.S1. If the list of lattice parameters proposed by the software contained reasonable solutions, they are indicated by a check mark, otherwise they are indicated by a cross. Crysfire2020 was used to compare the performance of these software. To evaluate the performance of each software without human intervention, all of the analysis were performed with the default settings.

Table S1: Comparison of indexing software on Park's benchmark materials

Material	Software					
	ITO[1]	FJZN[2]	TREOR[3]	KOHL[4]	DICVOL[5]	LZON[2]
$Ca_{1.5}Ba_{0.5}Si_5N_6O_3$	✓	×	×	×	×	×
$BaAlSi_4O_3N_5$:Eu^{2+}	×	✓	×	✓	✓	✓

3.2 Benchmark test using TREOR

We examined the performance of TREOR on our test data as a benchmark test. To achieve a fair comparison, we only consider crystal systems derived from lattice constants given by TREOR. 10,000 non-triclinic crystal structures randomly chosen from our test dataset were tested with TREOR90 redistributed with Crysfire2020. We processed output files of TREOR90 using `trlog`, also bundled with Crysfire2020, to summarise the raw outputs in a machine-readable format. Out of 10,000, more than half were somehow failed in the summarising process and we got best solutions for 4,413 crystal structures based on figure of merit. The achieved top-1 accuracy was 43.67%. The confusion matrix is shown in Fig.S4. The misidentification of trigonal and hexagonal systems to tetragonal system is noticeable, but the reason is uncertain.

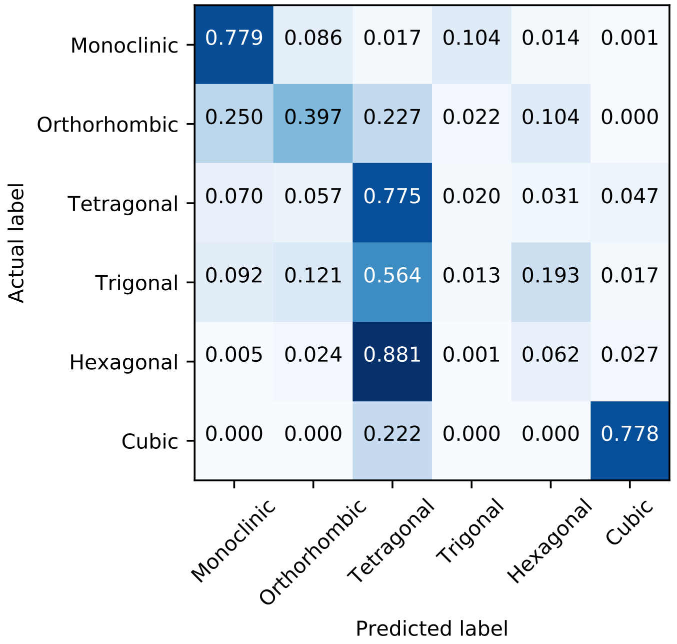

Figure S4: Confusion matrix of crystal system classification with TREOR which peak-indexing software

4 Testing with real diffraction data

Although our prediction models are not trained to overcome imperfections in real experimental data, showing the current prediction performance in real data would be helpful to readers. We used data from Samples 1-3 from the round-robin experiment organized by Le Bail *et al.* for the test data. The structures of these materials have been already determined in the study including complex ones. For more information on these materials, please see the project page for the round-robin study (`http://www.cristal.org/sdpdrr2/`).

The prediction results are shown in Tb. S2. Our method probably worked better than a guesswork for Samples 1 and 2, but it failed to predict the symmetry of Sample 3. Sample 3 has a complex structure with disordered fullerenes and Br, and it is mentioned that it took three years with many attempt to determine the structure. This result suggests that our method cannot replace long-lived software and trials-and-errors by experts at this moment, which is no surprise to us.

Table S2: Symmetry prediction results for Le Bail's benchmark materials

Material	Symmetry	Prediction (Top-10 for space groups)	Correct?
Sample 1	Monoclinic	**Monoclinic**	✓
($Al_2F_{10}[C_6N_4H_{20}]$)	P2/c (No. 13)	14, 2, 62, 19, 12, 29, 28, 58, 15, 138	×
Sample 2	Monoclinic	Orthorhombic	×
($Sr_5V_3(F/O/OH/H_2O)_{22}$)	P21/c (No. 14)	2, 15, 62, **14**, 176, 205, 138, 63, 4, 13	✓
Sample 3 ($C_{61}Br_2$)	Cubic	Orthorhombic	×
	I23 (No. 197)	12, 216, 51, 174, 200, 227, 175, 204, 187, 33	×

References

[1] Visser, J. W. A fully automatic program for finding the unit cell from powder data. *J Appl Crystallogr* **2**, 89–95 (1969).

[2] Shirley, R. A modified version of Visser's ITO zone-indexing program, using the Ishida & Watanabe PM criterion for zone evaluation. *Unpublished work* .

[3] Werner, P. E., Eriksson, L. & Westdahl, M. TREOR, a semi-exhaustive trial-and-error powder indexing program for all symmetries. *J Appl Crystallogr* **18**, 367–370 (1985).

[4] Kohlbeck, F. & Horl, E. M. Indexing program for powder patterns especially suitable for triclinic, monoclinic and orthorhombic lattices. *Journal of Applied Crystallography* **9**, 28–33 (1976).

[5] Boultif, A. & Louër, D. Indexing of powder diffraction patterns for low-symmetry lattices by the successive dichotomy method. *J Appl Crystallogr* **24**, 987–993 (1991).
